# Structure based pharmacophore modeling, virtual screening, molecular docking and ADMET approaches for identification of natural anti-cancer agents targeting XIAP protein

**DOI:** 10.1038/s41598-021-83626-x

**Published:** 2021-02-18

**Authors:** Firoz A. Dain Md Opo, Mohammed M. Rahman, Foysal Ahammad, Istiak Ahmed, Mohiuddin Ahmed Bhuiyan, Abdullah M. Asiri

**Affiliations:** 1 Department of Biomedical Science. College of Natural Sciences, Chosun University, Chosun, South Korea; 2 Department of Pharmacy, University of Asia Pacific, 74/A, Green Road, Farmgate, Dhaka, 1215 Bangladesh; 3 Department of Chemistry, Faculty of Science, King Abdulaziz University, Jeddah, 21589 Saudi Arabia; 4 Department of Genetic Engineering and Biotechnology, Faculty of Biological Science and Technology, Jashore University of Science and Technology, Jashore, 7408 Bangladesh; 5 Department of Chemistry, Jahangirnagar University, Savar Upazila, Dhaka, 1342 Bangladesh

**Keywords:** Biochemistry, Proteins

## Abstract

X-linked inhibitor of apoptosis protein (XIAP) is a member of inhibitor of apoptosis protein (IAP) family responsible for neutralizing the caspases-3, caspases-7, and caspases-9. Overexpression of the protein decreased the apoptosis process in the cell and resulting development of cancer. Different types of XIAP antagonists are generally used to repair the defective apoptosis process that can eliminate carcinoma from living bodies. The chemically synthesis compounds discovered till now as XIAP inhibitors exhibiting side effects, which is making difficulties during the treatment of chemotherapy. So, the study has design to identifying new natural compounds that are able to induce apoptosis by freeing up caspases and will be low toxic. To identify natural compound, a structure-based pharmacophore model to the protein active site cavity was generated following by virtual screening, molecular docking and molecular dynamics (MD) simulation. Initially, seven hit compounds were retrieved and based on molecular docking approach four compounds has chosen for further evaluation. To confirm stability of the selected drug candidate to the target protein the MD simulation approach were employed, which confirmed stability of the three compounds. Based on the finding, three newly obtained compounds namely Caucasicoside A (ZINC77257307), Polygalaxanthone III (ZINC247950187), and MCULE-9896837409 (ZINC107434573) may serve as lead compounds to fight against the treatment of XIAP related cancer, although further evaluation through wet lab is necessary to measure the efficacy of the compounds.

## Introduction

Hepatocellular carcinoma (HCC) is one of the most predominant type of primary liver cancer that has ranked fourth most common cause of cancer-related death worldwide^1,2^. Different factors are associated with the development of liver cancer including fatty liver disease, consumption of alcohol, hepatitis B and hepatitis C virus^3^. Early detection rate of the cancer is low and when the symptoms appear, cancer began to spread and be difficult to treat^4,5^. Liver transplantation, surgery, radiotherapy, chemotherapy are the most common approach to treat HCC, but in advance stage of cancer treatment failure rate are frequent^6^. To increase the survival rate early detection of liver cancer and better compounds, which are responsible to inhibit the growth of HCC is important to identify to reduce the HCC related disease.

Apoptosis is a type of programmed cell death that help to eliminate unwanted cells from multicellular organisms^7^. Apoptosis deficiency in the cells is an important cause of cancer that occur due to the IAPs. XIAP is one of the main anti-apoptotic proteins in the IAP family capable of neutralizing caspase-9 via BIR3 domain, where the effector caspases-3 and 7 is neutralize by BIR2 domain^8^. Cancer cells can escape from drug-induced death due to defects in pro-apoptotic death regulators or the presence of highly expressed pro-survival proteins, which is one of the main reasons for failure of chemotherapy. Oncogenes neutralization has shown the ability to reduce the lengthy process of chemotherapy and that is helpful to reduce the quantity and dose of drugs during cancer treatment. Therefore, XIAP based targeted would be an excellent treatment options for different cancer diseases including hepatocellular carcinoma^9^. Clinically antisense technology, SMAC-mimetics, siRNA has tried to decrease the overexpression of XIAP, but due to the neurotoxicity antisense based treatment (Example: AEG35156) were terminated in Phase-I clinical trial. The antisense based treatment process work based on reducing the XIAP mRNA level and increasing apoptotic cell death of stem cells. SMAC-mimetics are the most important compounds to neutralize the XIAP also work over the IAP families. Three amino acids present in the N terminal region of proline at the N-terminus of SMAC/Diablo are able to work over the binding groove of XIAP–BIR2 and XIAP–BIR3 domain at the same time^10^. Few important side effects have been observed in the case of developing of these compounds, SMAC/Diablo protein able to bind all BIR domains of several IAP most of are with cIAP1 and cIAP2 lead to the toxic effect or adverse effects due to the higher affinity of binding. To minimize adverse effects caused for higher binding affinity, antagonist molecules having the lower micromolar affinity to the XIAP-BIR2 domain is urgently need to develop^11,12^.

In-silico drug design consist of theoretical and computational approaches can be used to identify novel hits or leads against selected biological active macromolecules^13^. Nowadays, computer aided drug design (CADD) approach like pharmacophore modeling, virtual screening, molecular docking and dynamic simulation approaches are widely used to discover, develop, and analyze drugs and similar biologically active molecules^14^. In CADD approach, structure and ligand-based pharmacophore model can able to identify similar active molecules against specific target protein, where binding affinity of a large scale compound with target macromolecule can be evaluate easily by in-silico molecular docking process^15^. The biological activity of a compound can be evaluating, whenever the compound binds with targeted macromolecule and trigger a specific response. Calculation of binding capacity of a compound was time consuming and costly in conventional drug development due to require a large-scale in-vitro and in-vivo experiment^16^, in that case molecular docking approach make it easier within a short time. Pharmacokinetics and pharmacology properties like absorption, distribution, metabolism, and excretion (ADME) even toxicity of a compound can predict by using computer aided drug design process^17^. So, this study focused mainly on computer aided drug design process like structure-based pharmacophore modeling, virtual screening, ADMET, molecular docking and dynamic simulation approaches to identify the possible natural antagonist against XIAP protein to treat the cancer.

## Result and discussion

### Structure-based pharmacophore modeling and virtual screening

#### Pharmacophore model generation

XIAP is a nonredundant modulator of tumor necrosis factor-related apoptosis and best-defined anti-apoptotic IAP family member that directly neutralizes caspase-9 via its BIR3 domain. Over-expression of the protein is responsible for developing different cancers^7^. Chemical that has developed targeting XIAP are mostly toxic and has adverse effect. Therefore, natural compounds identical to the previously originated antagonist can be developed as a drug instead of chemically synthesis compound. Ten (10) chemically synthesis active antagonist of XIAP (Table 1) were collected through ChEMBL and advance literature search, which were docked with XIAP protein. The best binding score found for the antagonist Hydroxythio Acetildenafil (PubChem CID: 46781908) was − 6.8 kcal/mol, binding energy of other 9 molecules has shown in Table 1. Also, the interaction between XIAP protein and antagonist has provided in Table S1.

**Table 1:**
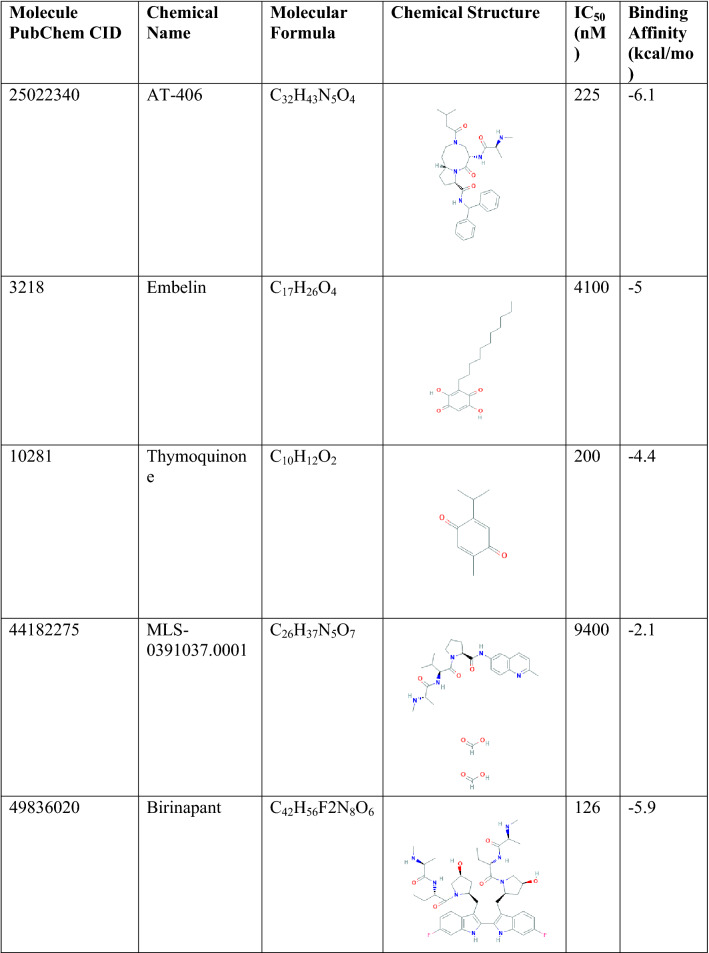

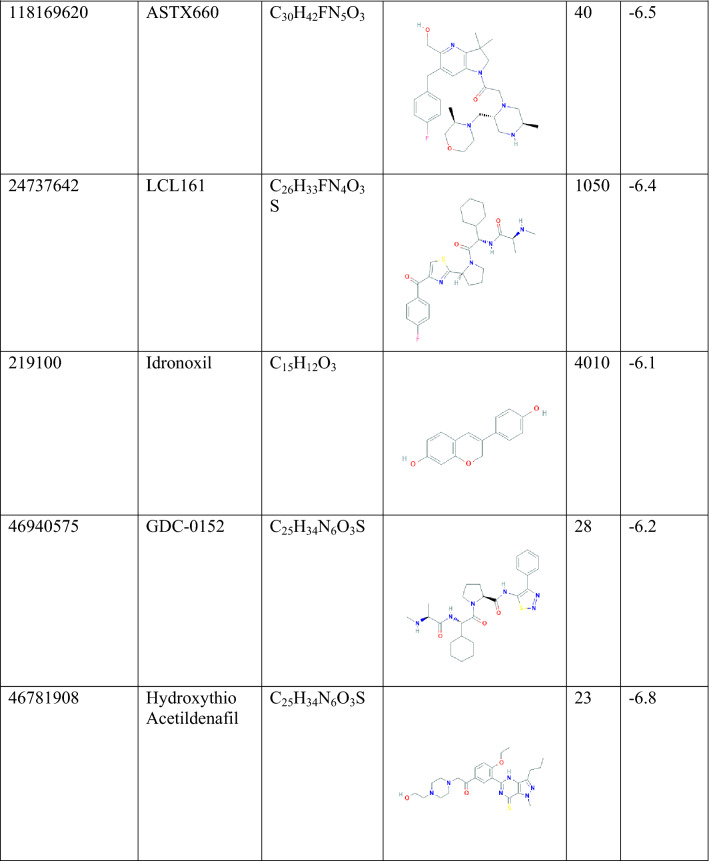
List of 10 known active antagonist of XIAP protein and their binding affinity towards the protein generated through molecular docking method.

For drug design, protein 3D structure determination is necessary and nowadays the most validated structure of protein can be mining from several protein data banks or homology modeling. To identify antagonist against desire protein crystal x-ray structure of XIAP protein (PDB: 5OQW) in complex with compound 46781908 recovered and a structure-based pharmacophore model to the enzymatic cavity was generated. Ligands binding capacity to the selected XIAP protein are determined experimentally and validated through x-ray diffraction method having IC_50_ value 40.0 nM^18^. The overall expression can be regulated by binding of the inhibitor to the active site of XIAP protein. Sometimes proper efficacy of inhibitor against any protein might not be reliable due to the improper binding. So, the determination of active series of inhibitors should be examined for sufficient interaction to get more biological activity compared to the existing one. LigandScout4.3 essential advance molecular design software was used to generate the key chemical features based on pharmacophore model.

**Figure 1:**
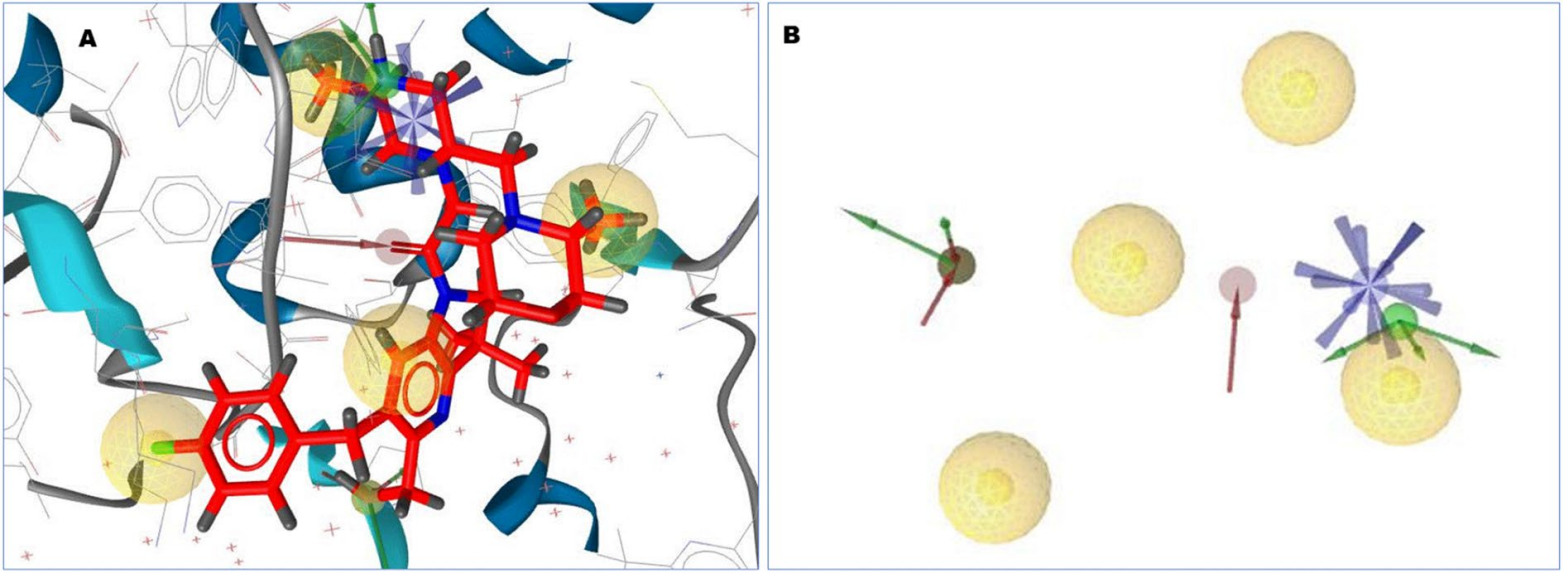
(**A**) The 3D structure-based pharmacophore model of XIAP protein in complex with 46781908 (CID) ligands derived from the X-ray derived crystal structure of XIAP protein (PDB ID: 5OQW). (**B**) Several pharmacophore features were generated after complex interaction, four yellow spherical shapes indicating hydrophobic interaction, one blue star shape depicting the positive ionizable with tolerance 2, three red colors arrow and spherical shapes indicating H bond acceptor having tolerance 1.5, five hydrogen bond donors represented by green spherical or arrow shape have been identified within the protein–ligand complex interaction. 15 exclusion volume, which were generated during pharmacophore modeling has not showed in this schematic presentation.

The different chemical features were determined, and total number was 14. Among theme four were hydrophobics, one positive ionizable bond, three H bond acceptor, 5 H bond donor, 15 exclusion volume features were presented as a protein ligand complex interaction (Fig. 1). To maintenance optimum pharmacophore features, some features have omitted during the time of pharmacophore model generation.

**Figure 2:**
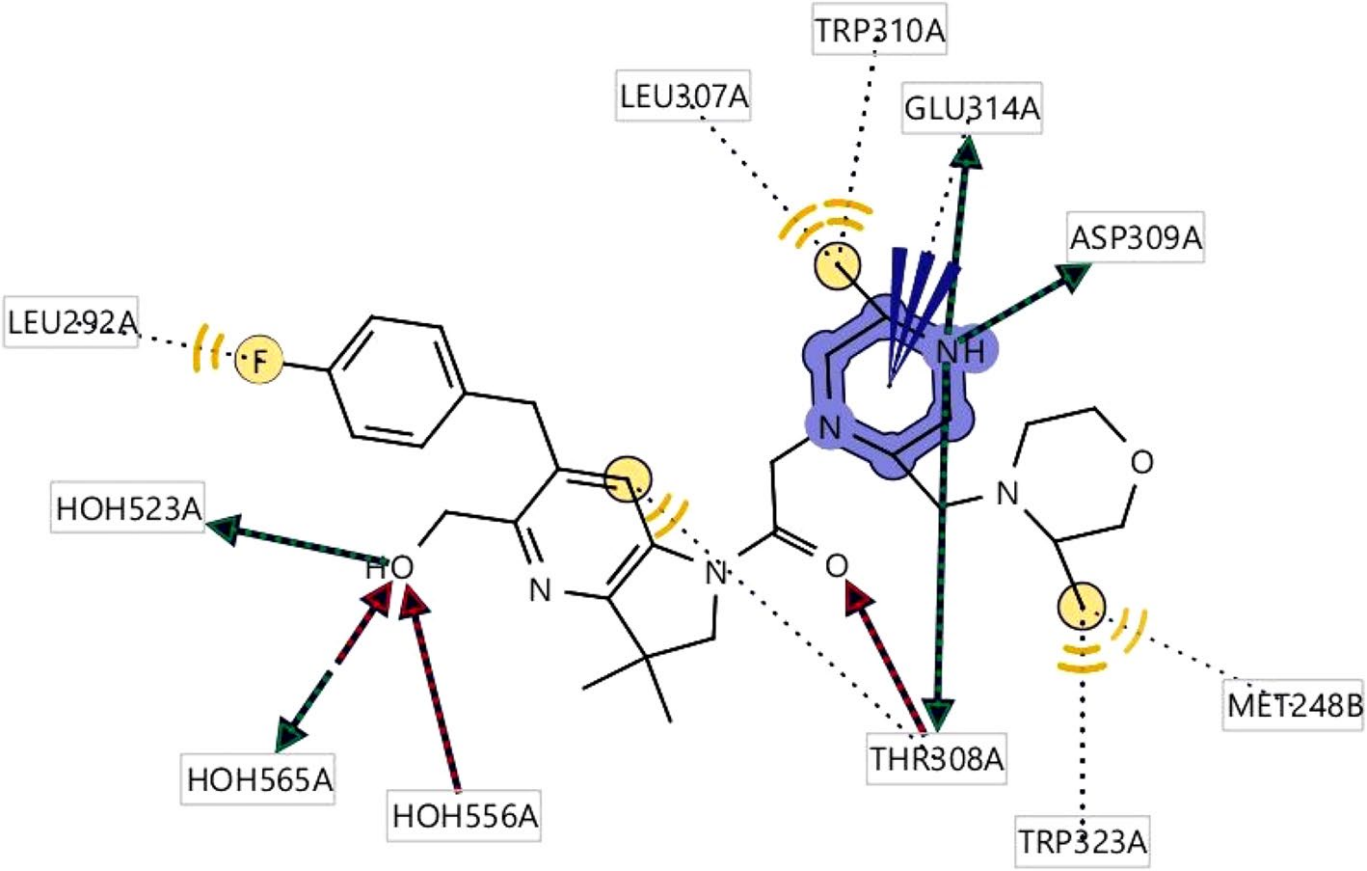
2D structure obtained during the pharmacophore modeling showing the hydrophobic interaction depicting yellow color and the interaction with the amino acid residues in our selected XIAP protein. Hydrogen bond donor (HBD) features most frequently participated in ligand–protein interaction were shown in green color, whereas the red color showing the interaction of Hydrogen bond acceptors (HBA) to the oxygen, nitrogen atom of the benzine ring and its different side chains. Hydrogen atoms as well as restricted area maintain the shape and position of the binding pocket did not mention in the figure.

Obtained pharmacophore features generated from the protein–ligand complex is depicted that hydrophobic interactions are predominant formed with the amino acid residues of the selected protein. HBD features have been found in several interactions with the protein, whereas nitrogen atoms in the benzine ring interacted with the THR308, ASP309, GLU314 amino acid (Fig. 2). One HBD was formed with the oxygen atom of the side chain of the amino benzine HOH523 number position. On the other site, HBD formation after ligand–protein interactions was shown as a red mark in the position of THR308, HOH556. HOH565. THR308, HOH556, which were bind to the oxygen atom, and nitrogen atom of the benzine ring bind to the HOH565. Partially two interactions such as HBD, HBA were formed also with the oxygen atom of HOH565. Positive ionizable pharmacophore features have also been found to be formed in GLU314 number position from the complex protein ligand structure.

#### Pharmacophore model validation

**Figure 3:**
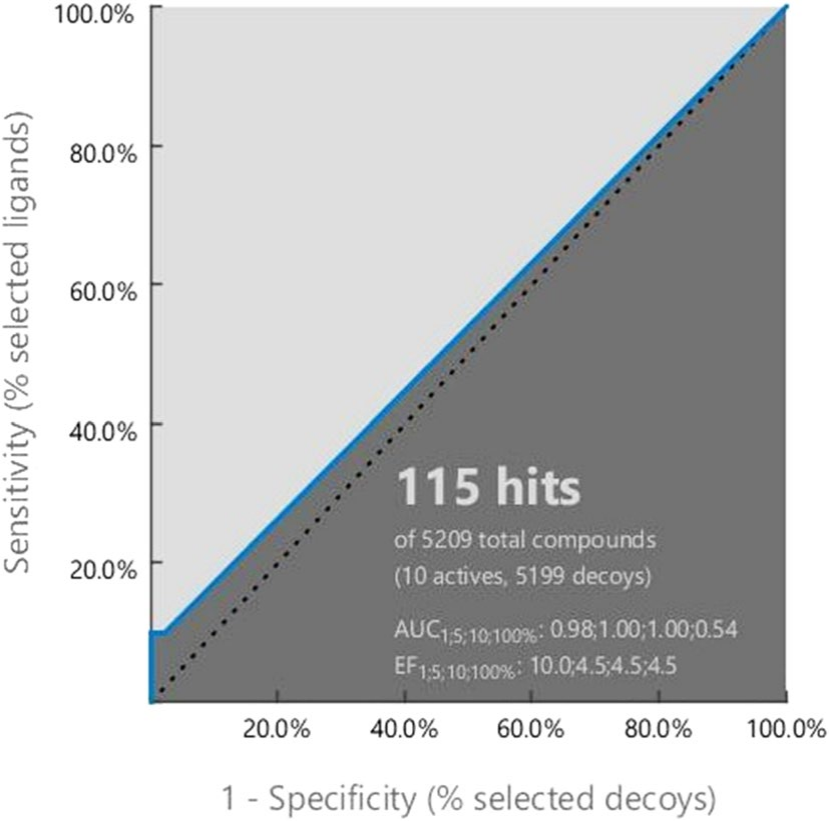
Receiver operating characteristic (ROC) curve generated based on the recognize ability of the active to decoy compounds of the structure-based pharmacophore model. The pharmacophore model was validated using a set of 10 XIAP active and 5199 decoy Compounds.

Validation is necessary to get the authentic pharmacophore analysis as well as to evaluate the quality of the molecular model^19^. Structure-based pharmacophore model generated in this study was validated before database screening to evaluate whether or not our models are capable to distinguish the active compounds from decoy set. The pharmacophore model was validated by using 10 actives (Table 1) known XIAP antagonists with correspondence 5199 decoy compound (Supplementary file) obtained from the enhanced Database of Useful Decoys (DUDe). The active test set with inhibitor constant IC_50_ values were merged with the decoy compounds and an initial screening was run to validate to model. The performance of a classification model like the AUC value and EF value of the compounds was estimated from the receiver operating characteristic curve (ROC). In general, ROC is a probability graph express the performance of a classification model that can give an idea about degree of separability, where AUC is used to describe the summary of the model performance. A model with higher AUC value should have better predictability^20^. The AUC value is ranging between 0 and 1, so the model whose prediction rate is 100% correct has an AUC value 1. In our validation process, the early enrichment factor (EF1%) was 10.0 with an excellent AUC (area under the ROC curve) value in 1% threshold was 0.98 (Fig. 3), which proved that our model has ability to distinguish true actives from decoy compounds.

#### Dataset generation for pharmacophore-base screening

Database generation is an important part for identification the best lead molecule during screening process. ZINC database is a curated collection of commercially available chemical compounds form which we can get the information regarding compound’s molecular weight, chemical structure, physical and chemical properties against biological active macromolecules. It contains more than 230 million purchasable compounds in 3D format to the freely accessible website, which is ready to dock^21^. It also provides information regarding various compound from different vendor like Ambinter as a natural compound database library. To create the database for pharmacophore based virtual screening, the previously obtained pharmacophore model generated for each active compound was submitted to ZINCPharmer^22^. Initially, it searches hits from the ZINC database of “ZINC natural products and ZINC natural derivatives” consist millions of Drug-like, Natural Products and FDA approved drugs. A maximum of 0.5 Å RMSD from sphere centers were used as input parameters for ZINCPharmer and a total 11,000 compounds was retrieved for further screening. The database of hit compounds from the ZINCPharmer were saved and downloaded for further screening.

#### Pharmacophore-based virtual screening

Pharmacophore interaction features generated from the protein–ligand complex was applied to the 11,000 natural compounds. During the screening process relative pharmacophore was used as a scoring function, where all query features were used as a screening mode and maximum four (4) features have omitted. It is difficult to match all of the query features during screening process that’s why some features have been omitted to increase the pharmacophore fit score. A higher score is desirable for optimum fitting with the desire environment than the compound will show better activity against the targeted macromolecules. A total seven hit compound with a fit score ranging from 94.75 to 105.31 were generated that matches all of the pharmacophore features. Usually, the pharmacophore fit value is shown the geometric fit of features to the 3D-structure-based pharmacophore model. The molecule with maximum fit score to the validated pharmacophore model should show activity against our desire XIAP protein. The compound, which remarked as hit was retrieved and saved for further evaluation.

## Molecular docking based virtual screening

### Binding site identification and receptor grid generation

**Figure 4:**
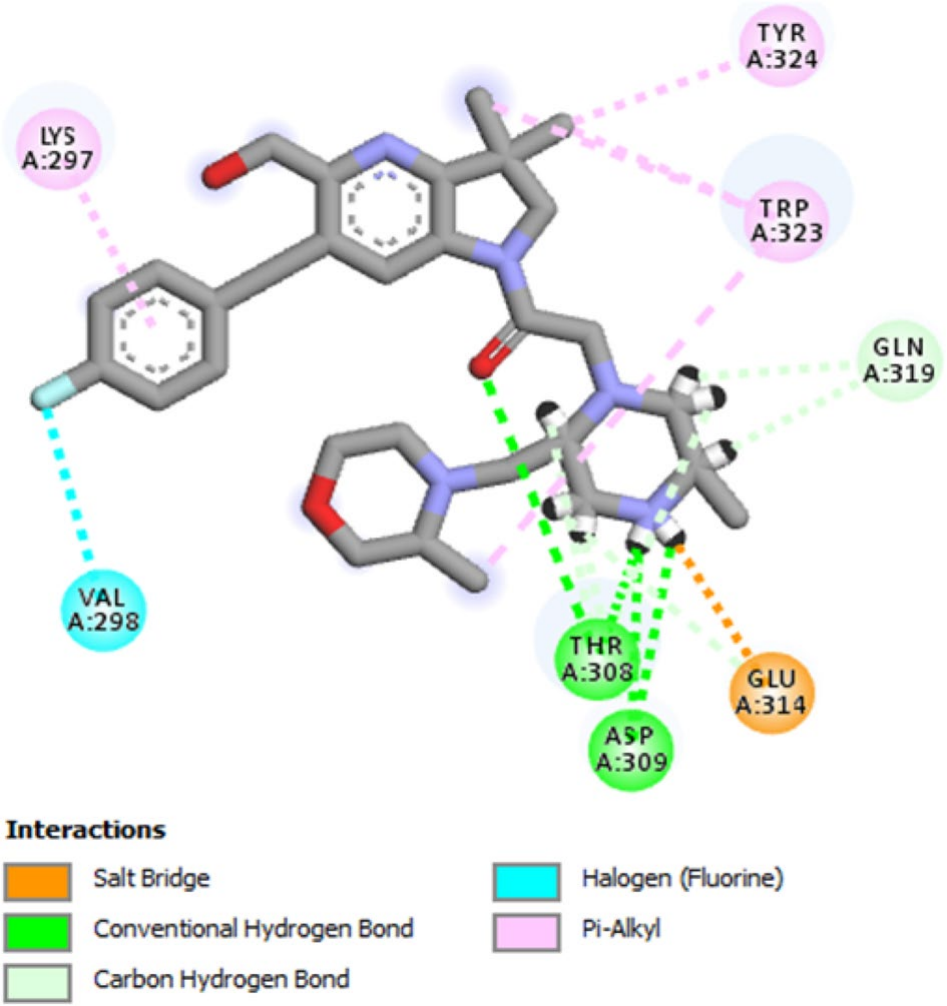
Depicted figure showing the binding site of the protein with the selected ligand complex (PDB ID: 5OQW). Several types or interaction were shown by color with different amino acids residues.

Based on the crystal structure, the desire XIAP (PDB ID: 5OQW) protein was bounded with three ligands. So, the protein pockets have different attachment site as well as shape for binding the favorable ligand. The binding position of the complex structure was retrieved so that the binding site can further utilize during molecular docking simulation. Analysis of the protein–ligand interaction revealed a salt bridge at the position of GLU314, four pi-alkyl bonds formed to formed at the benzine ring and side chain of benzine ring by interacting with TRP323, TYR324, LYS297. Four conventional hydrogen bonds were formed between the ASP309 and THR308 position. One halogen bond has observed at VAL298 with the fluorine attached to the benzine ring. At the same time, two carbon-hydrogen bonds were formed at GLN319 with the two sides of the same benzine ring (Fig. 4).

### Molecular docking

Molecular docking is an important part in drug design process, which is carried out in the study to evaluate the binding ability of the hits compounds to the target XIAP protein. XIAP monomeric proteins has decorated with two active chains such as A, B attached with three ligands (A4E, NA, ZN) with the protein. The protein was prepared and a receptor grid with box dimeter X = 30.06, Y = − 4.19 and Z = − 22.94 was generated depend on the previously obtained binding site to the chain A.

The specific number of drugs like hit (7) compounds were docked with XIAP by utilize PyRx tools Autodock vina to evaluate their binding capacity, which satisfied the characteristics of the pharmacophore model^23^. Among them, four compounds ZINC77257307, ZINC1070004335, ZINC247950187 and ZINC107434573 with binding affinity − 8.0 kcal/mol, − 7.8 kcal/mol, − 7.6 kcal/mol, and − 6.9 kcal/mol (Table 2), respectively shown better binding affinity than the XIAP antagonist CID: 46781908 (− 6.8 kcal/mol), that has used during main pharmacophore model generation. The binding affinity for all of hit shown in Table S2. Interestingly, compound which have higher pharmacophore fit score (Table 2) found higher docking score, and higher docking score indicates the better binding to the desire protein.

**Table 2:** Docking score with XIAP protein, pharmacophore fit score and the source of the top four selected compounds.

ZINC ID	Compound name	Docking Score (kcal/mol)	Pharm-Fit Score	Source of the compounds
ZINC77257307	Caucasicoside A	− 8.0	95.53	Helleborus caucasicus
ZINC1070004335	Venturicidin B	− 7.8	94.75	Streptomyces aureofaciens
ZINC247950187	POLYGALAXANTHONE III	− 7.6	95.70	Polygala tenuifolia
ZINC107434573	MCULE-9896837409	− 6.9	95.72	Unknown

### Interpretation of protein-ligands interactions

**Figure 5:**
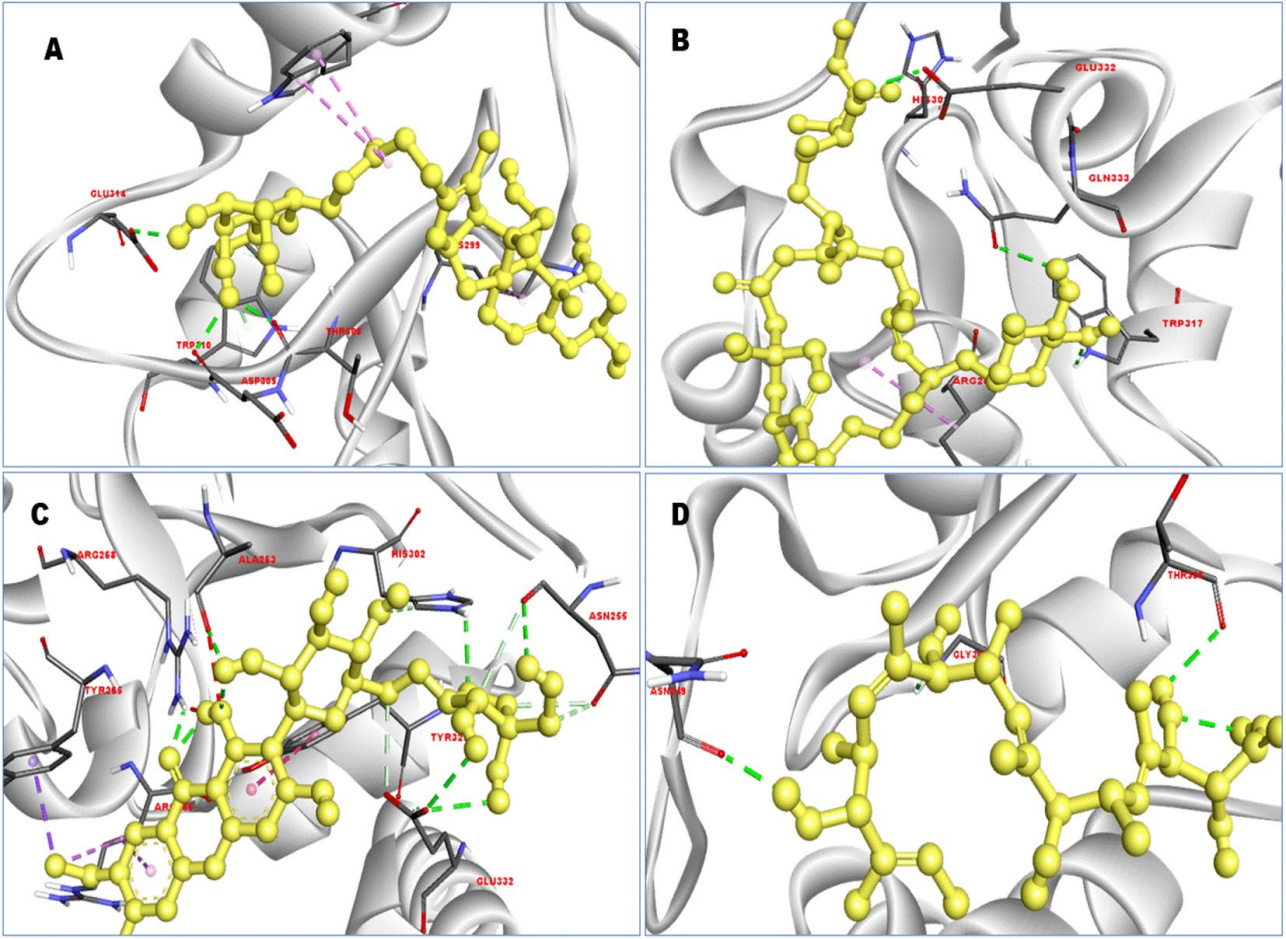
3D interaction between the protein–ligand complex. Here, figure (**A**) ZINC77257307, (**B**) ZINC1070004335, (**C**) ZINC247950187, and (**D**) ZINC107434573 showing the ligand contact with the protein XIAP after molecular docking.

**Figure 6:**
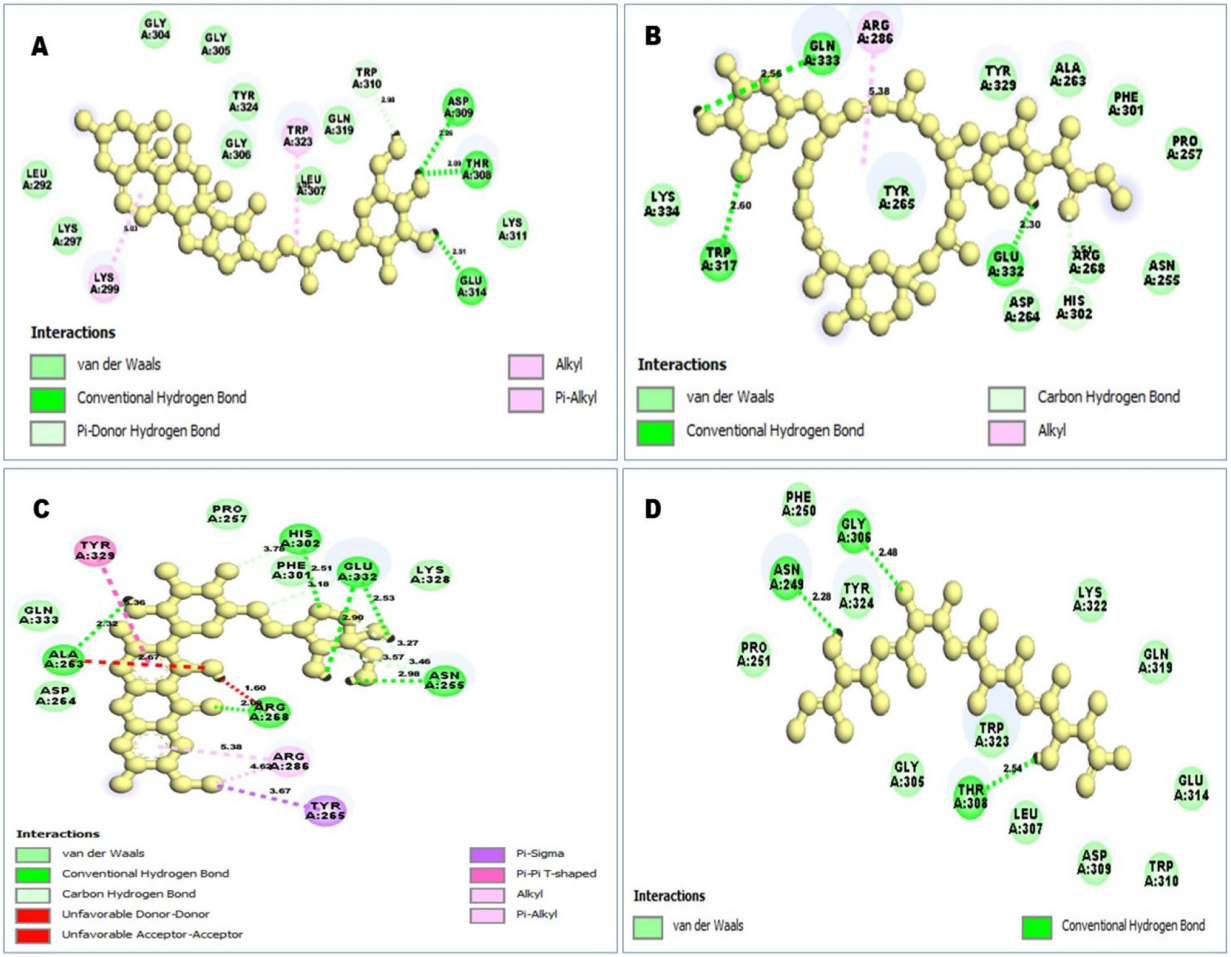
2D interaction between the protein–ligand complex. Here, figure (**A**) ZINC77257307, (**B**) ZINC1070004335, (**C**) ZINC247950187, and (**D**) ZINC107434573 showing the ligand contact with the protein XIAP after molecular docking. Different bonds types were described by different colors such as blue, red, purple, light pink, deep pink, green and sky blue.

Here, it is observed that compound that have better pharmacophore fit score gained better binding affinity ZINC77257307 (− 8.0 kcal/mol), ZINC1070004335 (− 7.8 kcal/mol), ZINC247950187 (− 7.6 kcal/mol) and ZINC107434573 in contrast to the compound CID: 46781908 (− 6.8 kcal/mol) showed in Table 2. ZINC77257307 formed nine van der walls interactions with LYS297, LEU292, GLY304, GLY305, TYR324, GLY306, LEU307, GLN319, LYS311, three conventional hydrogen bonds with ASP309, THR308, GLU314, one pi donor hydrogen bond with TRP310, one alkyl bond with LYS299, and two Pi-alkyl bonds with TRP323 with desire XIAP protein (Figs. 5 and 6).

In the case of ZINC1070004335 van der walls bond were predominantly formed with LYS334, TYR329, ALA263, PHE301, PRO257, ASN255, ARG268, ASP264, TYR265. The position of TRP317, GLN333, GLU332 acquired three conventional hydrogen bonds, one carbon-hydrogen bond at HIS302 and one alkyl bond with ARG286 position (Figs. 5 and 6).

For the compound ZINC247950187, the number of vans der walls interaction has decreased but increased other types of bond such as 5 van der walls interaction at position ASP264, GLN333, PRO257, PHE301, LYS328 has found to formed, which are less than the previous two compounds. Nine conventional hydrogen bonds with ALA263, ARG268, HIS302, GLU332, ASN255 position with four hydrogen atom, one pyridine, and one benzene ring with ARG268 and ALA263 position has found to formed (Figs. 5 and 6). For, ZINC107434573 it has observed to formed five carbon-hydrogen bonding in the position of ASN255, HIS302, GLU332, one pi-sigma with TYR265, one pi-pi t shaped bond with TYR329, one alkyl and pi alkyl bond with ARG286. The compounds also produced 11 van der walls bond with GLY305, PRO251, TYR324, PHE250, TRP323, LYS322, GLN319, GLU314, TRP310, ASP309, LEU307, and 3 conventional hydrogen bonds with ASN249, GLY306, THR308 position. The protein–ligand interaction mode for all the four compounds has listed in Table 3.

**Table 3:** Interaction result between the selected 4 ligands in complex with the protein XIAP.

Compounds ID	Conventional hydrogen bonds	Pi donor hydrogen bond	Alkyl bond	Carbon hydrogen bond	Unfavorable donor-donor bond	Unfavorable acceptor-acceptor	Pi-alkyl bond
ZINC77257307	ASP309 (2.26 Å), THR308 (2.09 Å), GLU314 (2.51 Å)	TRP310 (2.98 Å)	LYS299 (5.03 Å)				TRP323 (4.36 Å, 5.03 Å)
ZINC1070004335	TRP317 (2.6 Å), GLN333 (2.56 Å), GLU332 (2.30 Å)		ARG286 (5.38 Å)	HIS302 (3.51 Å)			
ZINC247950187	ALA263 (2.32 Å), ARG268 (2.08 Å), HIS302 (2.51 Å), GLU332 (2.53 Å, 2.90 Å), ASN255 (3.46 Å, 3.23 Å, 2.98 Å, 3.57 Å)	Pi-sigma	Pi-pi T shaped	ASN255, HIS302 (3.78 Å), GLU332 (3.18 Å)	ARG268 (1.60 Å)	ALA263 (2.67 Å)	ARG286 (4.62 Å, 5.38 Å)
		TYR265 (3.67 Å)	TYR329 (5.36 Å)				
ZINC107434573	ASN249 (2.28 Å), GLY306 (2.48 Å), THR308 (2.54 Å)						

### Pharmacophore features analysis

**Figure 7:**
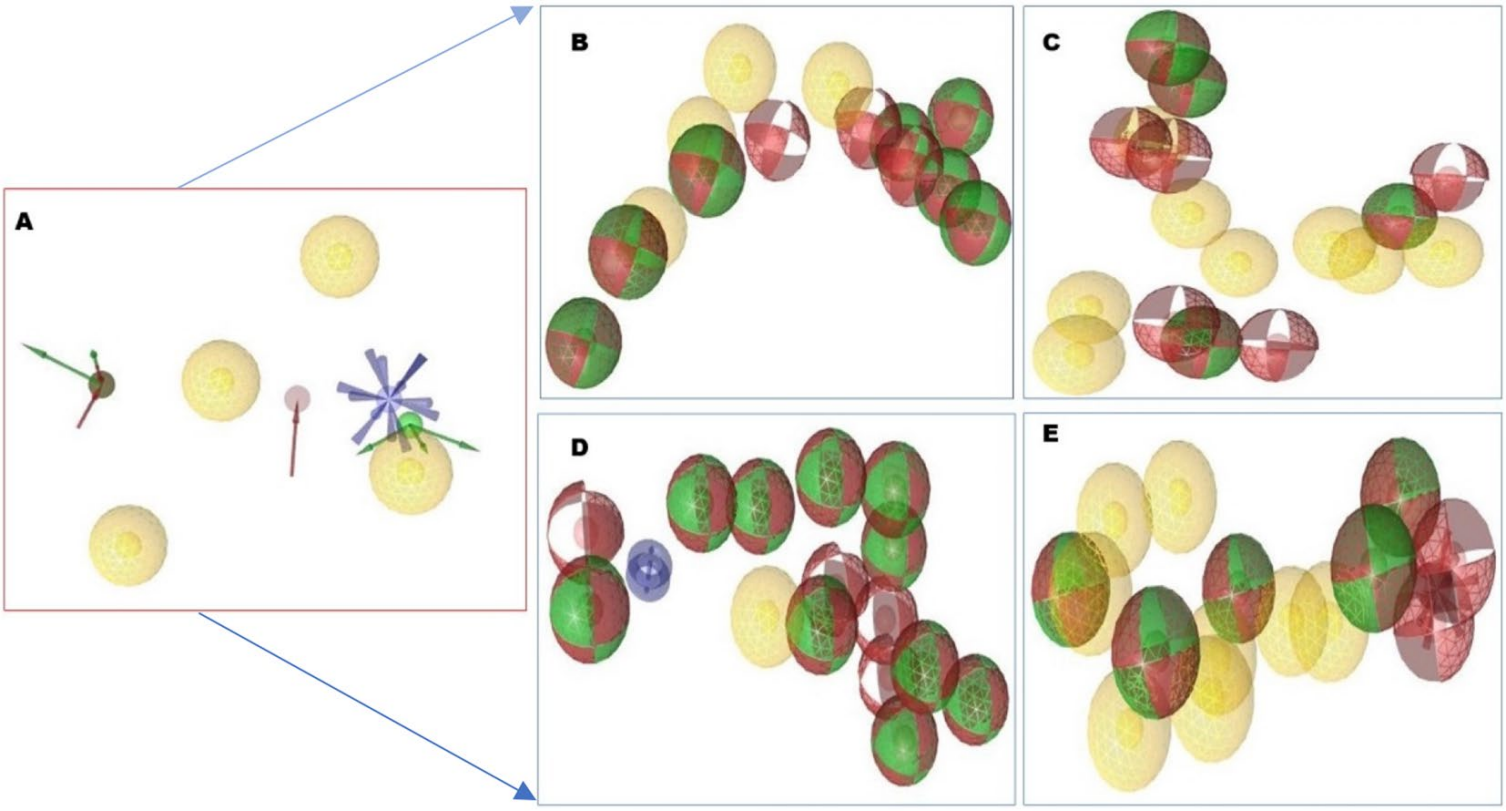
Pharmacophore features generated from the four selected compounds attach to the desire XIAP protein. Ligands attach to the protein (**A**) 46781908 had four hydrophobic (yellow), one positive ionizable (blue star), three H bond acceptor (red), and five hydrogen bond donors (green) pharmacophore features. Comparing to this most of our selected compounds (**B**) ZINC77257307, (**C**) ZINC1070004335, (**D**) ZINC247950187 and (**E**) ZINC107434573 have better pharmacophore features than antagonist 46781908.

Pharmacophore is a group of steric and electronic features that confirm optimal supramolecular interactions during virtual screening on large scale compound databases. It is powerful and more efficient method than molecular docking that can find molecules against specific target to induce or inhibit the macromolecular activity^24^. The compound which has similar or relevant properties should show the same or better activity like to the query compound. In this study, the top four compounds (based on docking score) ZINC77257307, ZINC1070004335, ZINC247950187 and ZINC107434573 pharmacophore features was analyzed and compared with the features of the antagonist 46781908. All of the compounds have better pharmacophore properties than the antagonist CID: 46781908, so these compounds should be effective to our target protein. The pharmacophore feature found of the four compounds has shown in Fig. 7.

## Absorption, distribution, metabolism and excretion (ADME) and toxicity test

### Analysis of ADME properties

After administration of the drug through any route to the human body or in the animal model, it undergoes the absorption, distribution, metabolism, excretion resulting active or passive transport to the target site^25^. Interaction with the target biological macromolecules might produce desirable or undesirable pharmacological effect. Drug design is a step-by-step evaluation process and lacking the evaluation my reason for rejection of the drug, which is costly for any companies. The bioavailability of a drug depends on the safety and efficacy, lack of safety and efficacy are the main cause of drug failure, which are mainly depend on the ADME properties. Here, we evaluate the ADME properties of the selected four compounds by using in silico SwissADME server to see the pharmacokinetic properties such as lipophilicity, water-solubility, drug-likeness, medicinal chemistry of the compounds^26^. The lipophilicity of compounds means they can easily diffuse through the cell membrane; hence the oral preparation is not suitable. Moreover, an injectable dosage form may be a better option to get a rapid onset of action as the gastrointestinal absorption is low. The ADME properties of the selected four compounds has shown in Table 4.

**Table 4:** List of pharmacokinetic properties (physico-chemical, lipophilicity, water solubility, drug likeness, and medicinal chemistry) of the selected 4 compounds.

Properties	Parameters	ZINC77257307	ZINC1070004335	ZINC247950187	ZINC107434573
Physico-chemical properties	MW (g/mol)	606.74 g/mol	706.95	568.48	454.60
	Heavy atoms	43	50	40	32
	Arom. heavy atoms	0	0	14	0
	Rotatable bonds	7	9	6	12
	H-bond acceptors	10	10	15	7
	H-bond donors	7	4	9	6
	Molar Refractivity	157.51	196.06	131.62	127.97
Lipophilicity	Log P_o/w_	1.58	4.60	3.53	2.87
Water solubility	Log S (ESOL)	Soluble	Poorly soluble	Soluble	Soluble
Pharmacokinetics	GI absorption	Low	Low	Low	Low
Drug likeness	Lipinski, Violation	2	1	2	1
Medi. chemistry	Synth. accessibility	7.82	9.66	5.95	5.96

### Analysis of toxicity

For better lead compound selection, in-silico toxicity measurement is an important procedure before drug candidate undergo clinical trial^25^. Computational based in-silico toxicity measurement has been widely used due to their accuracy, rapidity, accessibility, which can provide information about any synthesis or natural compounds. To identify the toxicity and adverse effect of the selected four compounds, we used both freely accessible TEST tool^27^, and ProTox-II server^28^. Each software was used to evaluate several toxicological parameters such as acute toxicity, hepatotoxicity, cytotoxicity, carcinogenicity, mutagenicity, immunotoxicity, and the result was achieved based on predicted median lethal dose (LD_50_) in mg/kg weight (Table 5). According to the ProTox-II server compound ZINC107434573 and ZINC247950187 both were belonging to class 4, LD_50_ range from 300 to 2000 mg/kg, these would be harmful in case of oral delivery. For ZINC1070004335, the LD_50_ value was less than < 50 mg/kg, so oral intake might be toxic or fetal, which were belongs to the toxicity class two in ProTox-II. ZINC77257307 was in the toxicity class 6 and the LD_50_ value was also more than 5000 mg/kg and it is also nontoxic but having some immunotoxicity.

**Table 5:** List of toxicity properties (Organ Toxicity, Toxicity Endpoints, Tox21-Nuclear receptor signaling pathways, Tox21-Stress response pathway, Fathead minnow LC_50_ (96 h), Developmental toxicity, Oral rat LD_50,_ Bioaccumulation factor_)_ of the selected 4 compounds.

Endpoint	Target	ZINC77257307	ZINC1070004335	ZINC247950187	ZINC107434573
Organ toxicity	Hepatotoxicity	Inactive	Inactive	Inactive	Inactive
Toxicity endpoints	Carcinogenicity	Inactive	Inactive	Inactive	Inactive
	Immunotoxicity	Active	Active	Active	Inactive
	Mutagenicity	Inactive	Inactive	Inactive	Inactive
	Cytotoxicity	Active	Inactive	Inactive	Inactive
	LD_50_ (mg/kg)	6000	50	1469	665
	Toxicity class	6	2	4	4
Tox21-Nuclear receptor signaling pathways	Androgen Receptor (AR)	Inactive	Inactive	Inactive	Inactive
	Aryl hydrocarbon Receptor (AhR)	Inactive	Inactive	Inactive	Inactive
Tox21-Stress response pathway	Heat shock factor response element	Inactive	Inactive	Inactive	Inactive
Fathead minnow LC50 (96 h)	mg/L	0.81	N/A	N/A	9.95E−02
48-h *Daphnia magna* LC_50_	mg/L	9.11	11.61	63.28	10.76
Developmental toxicity	value	0.47	0.53	0.39	0.36
Oral rat LD_50_	mg/kg	53.32	682.02	727.46	1127.01
Bioaccumulation factor	Log10	1.57	N/A	1.20	− 0.33

## Molecular dynamics (MD) simulation

MD simulation is used to explore the binding stability of protein–ligand docking complexes^29^. The MD simulation also provide information regarding intermolecular interaction within a reference time. Herein, the complexes docking file of selected four natural compounds and one reference antagonist bind with XIAP protein were analyzed by utilize MD simulation approaches to confirm the stability and intermolecular interactions between protein and molecules against 50 ns time interval. Trajectories of MD were extracted by utilize SID in Maestro-Desmond interface and the simulation result has described based on RMSD, RMSF and Protein–Ligand (P–L) interaction mapping.

### RMSD analysis

Root mean square deviation (RMSD) in MD simulation is used to measure the average distance generated by displacement of a selected atoms for a specific time frame with respect to a reference time frame^14^. Initially, RMSD value of specific protein structure such as Cα, backbone, sidechain and heavy atoms are computed, after that RMSD of the protein fit ligand from all the time frames during the reference time (in our case 50 ns) is calculated. RMSD for frame x can be calculated from the following equation (Eq. 1).

1 $$RMSD_{x} = \sqrt {\frac{1}{N}} \sum\limits_{{{\text{i}} = 1}}^{N} {(r_{i}^{\prime } (t_{x} )) - (r_{i} (t_{{ref}} ))^{2} }$$

Here, *N* can define as the number of atoms in the atom selection; *tref* is the reference time, and *r'* define the location of the selected atoms within the frame *x* after superimposing on the reference frame, *tx* expressed the recoding intervals of x.

#### RMSD of protein

**Figure 8:**
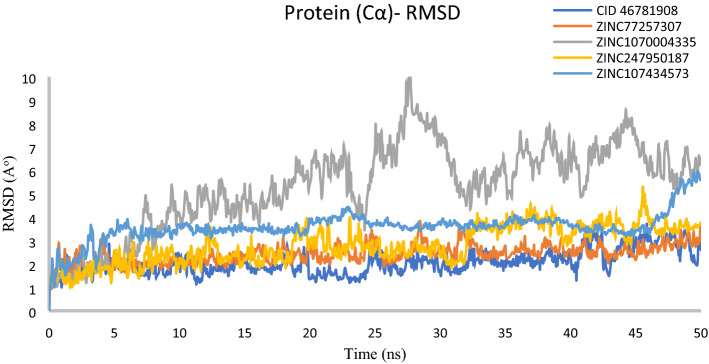
RMSD values extracted from Cα of the protein–ligand docked complexes, viz CID: 46781908 (Gray), ZINC77257307(orange), ZINC1070004335, ZINC247950187 (Gold), ZINC107434573 (Blue), with respect to 50 ns simulation time.

Based on RMSD result, it can be determined that simulation has equilibrated or not. Fluctuations between 1–3 Å within a reference protein structure is perfectly acceptable, where much larger value indicate large conformational change of the protein and the system is not stable. Analysis of our four protein–ligand docking complex found Cα atoms of XIAP showed acceptable fluctuations < 3 Å, except in XIAP- ZINC1070004335 complex. The compound ZINC1070004335 exhibited an extended variation 5.1 Å and a maximum fluctuation 8.81 Å (between 26 and 28 ns) observed during 50 ns simulation run (Fig. 8). From the data we can assume that XIAP undergo protein conformation changes influence by the binding of ZINC1070004335.

#### RMSD of ligand

**Figure 9:**
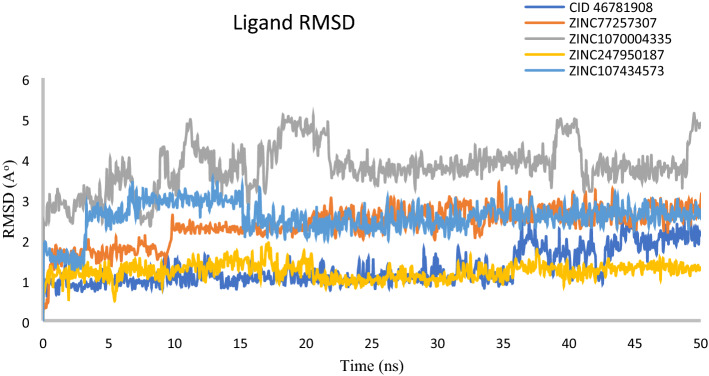
RMSD values extracted from protein fit ligand of the protein–ligand docked complexes, viz CID: 46781908 (Gray), ZINC77257307(orange), ZINC1070004335, ZINC247950187 (Gold), ZINC107434573 (Blue), with respect to 50 ns simulation time.

Furthermore, analysis of RMSD from the data obtained from protein fit ligands showed minimum variations (< 3 Å), except for ZINC1070004335 complex (> 4.84 Å) at the end of 50 ns simulation interval (Fig. 9). However, it is necessary to mention that three natural compound ZINC77257307, ZINC247950187, ZINC107434573 and the chemical antagonist CID: 46781908 docked with XIAP exhibited equilibrium within 10 to 15 ns, with an exception for XIAP- ZINC1070004335 complex, which has tried to exhibited state of equilibrium after 25 ns.

### RMSF analysis

The Root Mean Square Fluctuation (RMSF) is necessarily important for characterization and determination the local conformational change in the protein chain and the compounds utilized as ligand^14^. The RMSF of the residue *i* can be calculated by the following equation (Eq. 2).

2 $$RMSF_{i} = \sqrt {\frac{1}{T}} \sum\limits_{{{\text{t}} = 1}}^{T} {\left\langle {(r_{i}^{\prime } (t)) - \left( {r_{i} \left( {t_{{ref}} } \right)} \right)^{2} } \right\rangle }$$

Here, *T* can define as the trajectory time; *tref* is the reference time, and *r'* define the location of the selected atoms within the residue *i* after superimposing on the reference frame, and (< >) expressed the average of the square distance taken over residue b.

**Figure 10:**
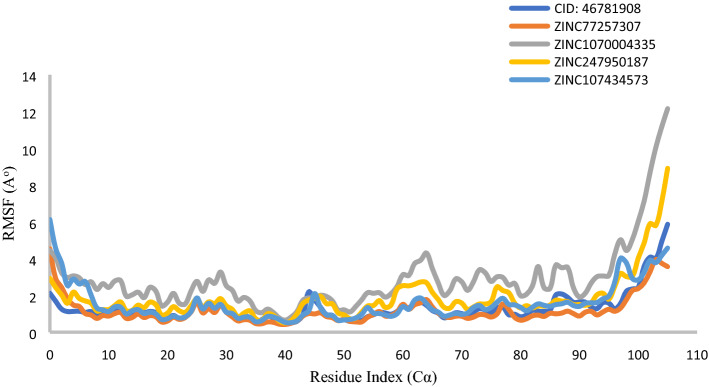
RMSF values extracted from protein Cα atoms, viz CID: 46781908 (Gray), ZINC77257307(orange), ZINC1070004335, ZINC247950187 (Gold), ZINC107434573 (Blue), with respect to 50 ns simulation time.

The local structural fluctuations of XIAP protein in complex with natural compound were calculated by using the deviations contributed by residues index Cα. Interestingly, residues for all protein have found a minimum RMSF values, except in N-terminal minimum 4.18 Å to maximum 12.16 Å (Fig. 10). So, analysis of RMSF and RMSD value for all protein–ligand complex supported the combined screened potential compounds except compound ZINC1070004335 against XIAP protein.

### Protein–ligand interaction analysis

**Figure 11:**
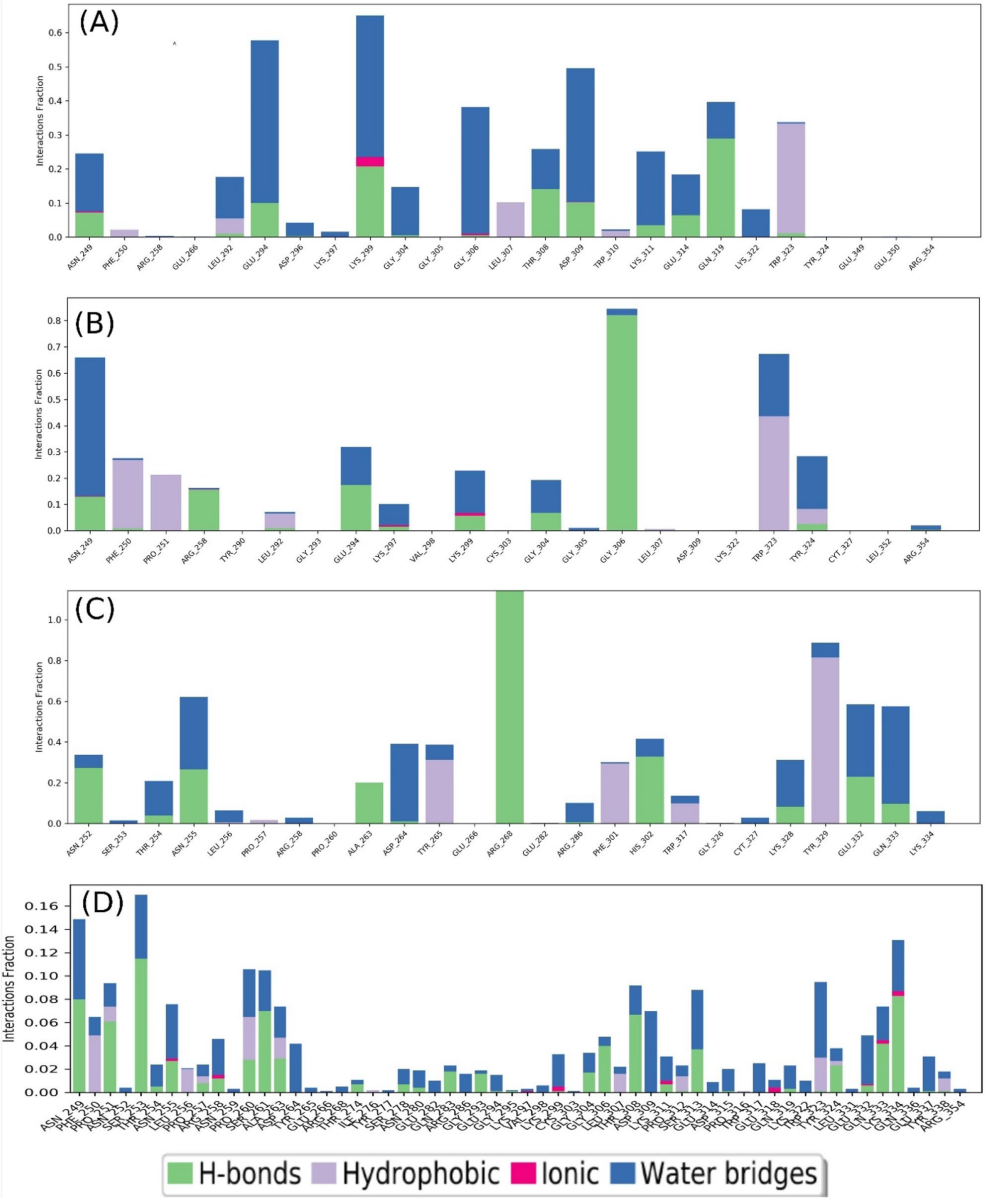
Protein–ligand contact mapping for XIAP with potential natural compounds, i.e. (**A**) ZINC77257307, (**B**) ZINC1070004335, (**C**) ZINC247950187, and (**D**) ZINC107434573 extracted from 50 ns MD simulations.

Protein–ligand contact occur through hydrogen bonding, ionic bonding, water bridges and hydrophobic bonding play an important role to design a molecule into effective drug. Protein–ligand contact occurred to XIAP protein and selected four natural compounds were analyzed from the MD trajectories by using default parameters of Desmond module. All the selected natural compounds ZINC77257307, ZINC247950187, ZINC107434573 and ZINC1070004335 showed tangible contact with most of the protein residues (Fig. 11), i.e., ASN 249 and LYS 299 except compound ZINC247950187 but maintain optimum binding with other residue like GLU 332 of the XIAP protein. Interestingly, the residue GLU332 were also found in the respective ligand–protein complex docking structure of the selected compounds (Table 3). Moreover, the four compounds screened through different filtering process exhibited considerable intermolecular interaction.

## Conclusions

In this in-silico approach, three new natural compound ZINC77257307, ZINC247950187, and ZINC107434573 have discovered that may be able to induce apoptosis through freeing up caspases. These selected compounds have a higher binding affinity ranging between − 6.9 and − 8.0 kcal/mol with desire XIAP protein. Based on the in-silico toxicity test, they have found a lower toxicity, and ADME analysis determined the easily absorbability to the tissue site, which is readily fat soluble. Initially, a structure-based pharmacophore model was developed following by virtual screening, molecular docking, ADMET analysis and MD simulation. Four compounds were reached at the last step until MD simulation, but stability of the compound ZINC1070004335 was unfavorable to the protein XIAP in MD simulation, which has rejected. The top three natural compound that exist during the A-to-Z virtual screening process may serve as lead molecules to fight against cancer.

## Material and methods

### Structure-based pharmacophore modeling and virtual screening

#### Structure-based pharmacophore modeling

The active antagonists of X-linked inhibitor of apoptosis protein (XIAP) were generated by collecting all available target annotations from ChEMBL (on the basis of high-confidence activity data) and extensive literature search^30^. In order to generate a structure-based pharmacophore models, the 10 active antagonists (Table 1) obtained from ChEMBL (https://www.ebi.ac.uk/chembl/) and literature search have docked with the XIAP (PDB ID: 5OQW) protein by utilizing the PyRx AutoDock Vina option based on scoring functions. The best compound with highest binding affinity (kcal/mol) was selected for structure-based pharmacophore modeling. Top scoring compound in complex with XIAP protein was used to interact with the natural compounds resulting retrieval of hits. LigandScout 4.3 advance software was used to produce a structure-based pharmacophore model^31^. This advanced software works by making the interaction between inhibitors and critical amino acids of the active sites in our target protein. This software interprets ligand-receptor interaction with different pharmacophore features such as hydrogen bond donor, charge transfer, hydrophilic and hydrophobic regions, and hydrogen bond acceptors. We have detected other features using stepwise algorithms such as the number of aromatic rings, hybridization state, the pattern of binding, the distance of receptor molecules^32^. For identify better and optimum compound structure, we deleted hydrophilic properties from the protein by using ligand scout, excluded or included features to the active site necessary to maintain sterically circumference of the macromolecule.

#### Pharmacophore model validation

Pharmacophore validation helps to evaluate the potential features of active and inactive compounds usually can be obtained from specific protein–ligand interaction^33^. Pharmacophore model generated from the protein–ligand complex was validated for its performance to distinguish active compounds from decoys by screening a set of 10 known actives and correspondence 5199 decoy correspondence obtained from DUD-E decoys database^34^. The ten active antagonists obtained from ChEMBL (https://www.ebi.ac.uk/chembl/) was remarked as “active” against XIAP, which was experimentally validated that’s why the compounds have chosen for further experiment. The database from DUD-E was converted in the .ldb format before screening by the “create screening database” menu of LigandScout 4.3^31^. Here, it has been assessed the quality of the structure-based model based on the GH score and early enrichment factor (EF).

#### Dataset Generation for pharmacophore-base screening

It can be identified the structurally novel and active molecules by the completion of virtual screening depending on the generated pharmacophore model^35^. ZINC (https://zinc.docking.org/) is a freely available chemical database, which is being utilized to identify the potential lead compounds^21^. Compounds from the database can be searched depending on the structure, name of the compound, or using the chemical smile ID. Physical and chemical properties such as 2D and 3D structure determination, the boiling point, the melting point were analyzed. Molecular weight, crystal structure, and biological application information can be also obtained of the desired compound. In the case of the desired compound, it has given priority, the compound having the most similar features matches the required pharmacophore features and can easily interact with our target protein. It has been chosen the possible hit compounds whose maximum features were matched to query pharmacophore. For the study, initially we have screened ZINC natural product library by using ZINCPharmer (http://zincpharmer.csb.pitt.edu/pharmer.html) server for target XIAP based on pharmacophore features^22^.

#### Pharmacophore-based virtual screening

Database generated from ZINCPharmer was subject to screen against the validated structural based pharmacophores features. LigandScout 4.3 advanced help to make and getting a 3D model in case of protein–ligand interaction and worked to change compounds into the specific (idb) file format. These compounds were passed directly into the database list for quick pharmacophore features based virtual screening. The securitizing has been completed by selecting relative pharmacophore-fit as a getting few functions based on omitting some features more than 2. Fitted hit compounds were arranged based on the pharmacophore fit score and subject to further validation.

## Molecular docking based virtual screening

### Protein and ligand preparation

Protein preparation in computational biology is a process by which macromolecular structure are converted into more suitable form for a computational experiment^36^. Prior to docking crystal structures of protein is needed to prepare which are not part of the x-ray crystal structure refinement process such as addition and optimization hydrogen bonds, remove atomic clashes, and perform other operations. For the study, desired 3D structure of XIAP protein was obtained from the protein data bank (PDB ID: 5OQW), which was determined experimentally and validated through X-ray diffraction method having resolution 2.31 Å and R-value free score 0.246 that is significantly less than standard value 0.25. The X-ray crystallography structure of our desire protein was prepared by the following steps (i) water, metal ion and cofactors were removed, (ii) polar hydrogen bond was added and non-polar H was merged and (iii) gasteiger charges were calculated by using AutoDockTools (ADT) 1.5.6^36,37^. Selected hit compounds reterived from Ligandscout was prepared and the energy was minimized and bond angle was opitimized by default of the Universal Force Field (UFF) for each ligand.

### Active site identification and grid generation

Binding of ligand or drug molecules to the specific site of protein is the key strategy to treat a particular disease. Improper attachment of ligand may show several side effects in the body with higher possibities of toxicities also^38^. These bininding affinities depend on several features H bond donars, hydropohobic or hydrophilic interaction, ionization, chelation of zinc compound. In the study, we used BIOVA Discovery Studio Visualizer Tool 16.1.0 to find the binding site of our desire protein. Moreover PrankWeb (https://prankweb.cz/) server was used to analyze the all probable binding site of the desire protein structure^39^. The server utilizes novel machine learning-based method for prediction of ligand binding sites from protein structure. Receptor grid was generated after selection of the active site of protein by using the PyRx software.

### Molecular docking

Selected hits compounds obtained by pharmacophore screening were subject to molecular docking, which was carried out by PyRx virtual screening software. In computational biology, PyRx is utilize as a virtual screening software that has identified many potential drug candidate against several diseases^40^. The software includes both AutoDock and AutoDock Vina with the Lamarckian genetic algorithm (LGA) as scoring function. This study used PyRx tools AutoDock Vina to proceed the molecular docking interactions. Resultant docked compound with better binding affinity (kcal/mol) were retrieved and visualized by using BIOVA Discovery Studio Visualizer Tool 16.1.0.^41^.

## Absorption, distribution, metabolism and excretion (ADME) and toxicity test

### Absorption, distribution, metabolism and excretion (ADME)

Evaluation of Absorption, Distribution, Metabolism and Excretion (ADME) properties is one of the major criteria before developing molecule into a drug^29,42^. Previously many drug candidate could not fit the clinical trial demand and so the computer-based prediction is important for the early stage of prediction. Physiochemical properties, hydrophobicity, lipophilicity, gastrointestinal environment, blood brain barrier are directly affected by the ADME profile before the excretion of drug from body through urine and feces^43^. The freely accessible Swiss-ADME (http://www.swissadme.ch/) server was used to evaluate the ADME properties such as solubility profile, GIT absorption, bioavailability profile of the selected compounds^26^.

### Toxicity test

Computational based approach has made it possible to measure the toxicity by in silico methods for accessing the safety profile of the desired compounds. Otherwise, these compounds may show the harmful effect on human and animals. Toxicity profile can evaluate and determine the mutagenicity, carcinogenicity, LD_50_ value, immunotoxicity in both quantitatively and qualitatively^44^. Toxicity Estimation Software Tool (TEST) is freely accessible software, which was used in this study to estimate toxicity of our compounds without requiring any other external programs^27^. The toxicity estimator TEST tools are being used for selective molecules that is work based on Quantitative Structure–Activity Relationships (QSARs) methodologies. Additionally, ProTox-II (http://tox.charite.de/protox_II) server was used to determine the toxic effect of the selected four compounds^28^. Different toxicological pathways including nuclear receptor signaling pathways, stress response pathways can be obtained from this site.

## Molecular dynamics (MD) simulation

### Explicit-solvent MD simulation

In order to further evaluate of protein toward the binding mode of our candidate molecules, the best poses obtained from re-docking studies were evaluated through 50 ns molecular dynamics (MD) simulations for measuring the complex stability. The MD simulations was carried out by using Desmond module in Schrödinger Release 2020-3 (Academic version) suite under Linux environment^[÷45^. The complex protein–ligand interaction was first solvated with simple point charge (SPC) water model with boundary condition orthorhombic box shape. Buffer box calculation method with box distance 15 Å (a = 5 Å, b = 5 Å, and c = 5 Å) on both side for all of the complex’s atoms has assigned. The system was neutralized by adding Na^+^ and Cl^-^ with a salt concentration 0.15 M. NPT ensemble was performed at constant pressure (1.01325 bar) and temperature (300 K) with recoding intervals 50 ps with energy 1.2, where OPLS-2005 force field was utilized to carried out the MD simulation.

### Post-dynamics trajectory analysis

The trajectories generated after completing the MD simulation were further analyzed by using Simulation Interaction Diagram (SID) of Desmond module in Schrödinger package. Based on the simulation’s trajectories, the stability of the ligand–protein complexes was determined according root-mean-square deviation (RMSD), root-mean-square fluctuation (RMSF) and Protein–Ligand contacts.

## Supplementary Information

Supplementary Tables.
