# Author Correction: Structure based pharmacophore modeling, virtual screening, molecular docking and ADMET approaches for identification of natural anti-cancer agents targeting XIAP protein

**DOI:** 10.1038/s41598-021-97945-6

**Published:** 2021-09-21

**Authors:** Firoz A. Dain Md Opo, Mohammed M. Rahman, Foysal Ahammad, Istiak Ahmed, Mohiuddin Ahmed Bhuiyan, Abdullah M. Asiri

**Affiliations:** 1 Department of Biomedical Science, College of Natural Sciences, Chosun University, Chosun, South Korea; 2 Department of Pharmacy, University of Asia Pacific, 74/A, Green Road, Farmgate, Dhaka, 1215 Bangladesh; 3 Department of Chemistry, Faculty of Science, King Abdulaziz University, Jeddah, 21589 Saudi Arabia; 4 Department of Genetic Engineering and Biotechnology, Faculty of Biological Science and Technology, Jashore University of Science and Technology, Jashore, 7408 Bangladesh; 5 Department of Chemistry, Jahangirnagar University, Savar Upazila, Dhaka, 1342 Bangladesh

Correction to: *Scientific Reports* 10.1038/s41598-021-83626-x, published online 18 February 2021

In the original version of this Article Abdullah M. Asiri was incorrectly affiliated with ‘Department of Pharmacy, University of Asia Pacific, 74/A, Green Road, Farmgate, Dhaka, 1215, Bangladesh’. The correct affiliation is listed below.

Department of Chemistry, Faculty of Science, King Abdulaziz University, Jeddah 21589, Saudi Arabia

The original Article and accompanying Supplementary Information file have been corrected.

