# Supplementary material for: Structure based pharmacophore modeling, virtual screening, molecular docking and ADMET approaches for identification of natural anti-cancer agents targeting XIAP protein
Source: Sci Rep. 2021 Feb 18;11:4049. doi: 10.1038/s41598-021-83626-x (PMC7892887; doi:10.1038/s41598-021-83626-x)
Supplement: Supplementary file 1 — Supplementary Tables. [file 41598_2021_83626_MOESM1_ESM.docx]

**Structure based pharmacophore modeling, virtual screening, molecular docking and ADMET approaches for identification of natural anti-cancer agents targeting XIAP protein**

Firoz A. Dain Md Opo ^1,2^, Mohammed M. Rahman ^3, *^, Foysal Ahammad ^4^, Istiak Ahmed ^5^, Mohiuddin Ahmed Bhuiyan ^2^, Abdullah M. Asiri ^3^

^1^Department of Biomedical Science. College of Natural Sciences, Chosun University, Chosun, South Korea

^2^Department of Pharmacy, University of Asia Pacific, 74/A, Green Road, Farmgate, Dhaka-1215, Bangladesh.

^3^Department of Chemistry, Faculty of Science, King Abdulaziz University, Jeddah 21589, Saudi Arabia

^4^Department of Genetic Engineering and Biotechnology, Faculty of Biological Science and Technology, Jashore University of Science and Technology, Jashore, 7408, Bangladesh

^5^Department of Chemistry, Jahangirnagar University, Savar Upazila, Dhaka-1342, Bangladesh

***Corresponding author:** [mmrahman@kau.edu.sa](mailto:mmrahman@kau.edu.sa) (M.M. Rahman)

Table S1: Showing the interaction between XIAP (PDBID: 5OQW) and selected active antagonists.

| **PubChem CID** | **Interaction residues** | **3D Interaction** |
| --- | --- | --- |
| **46940575** | **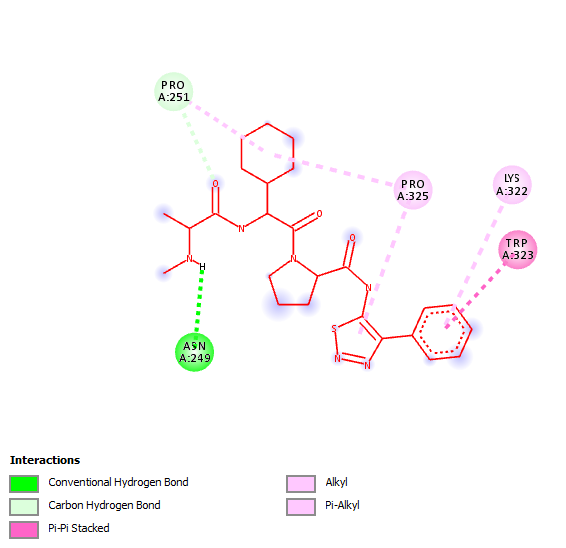** | **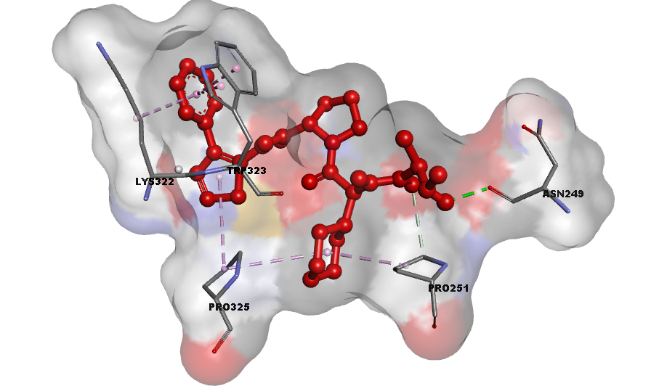** |
| **3218** | **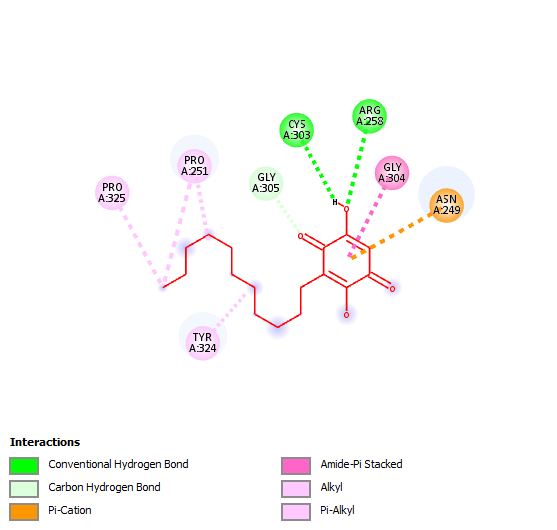** | **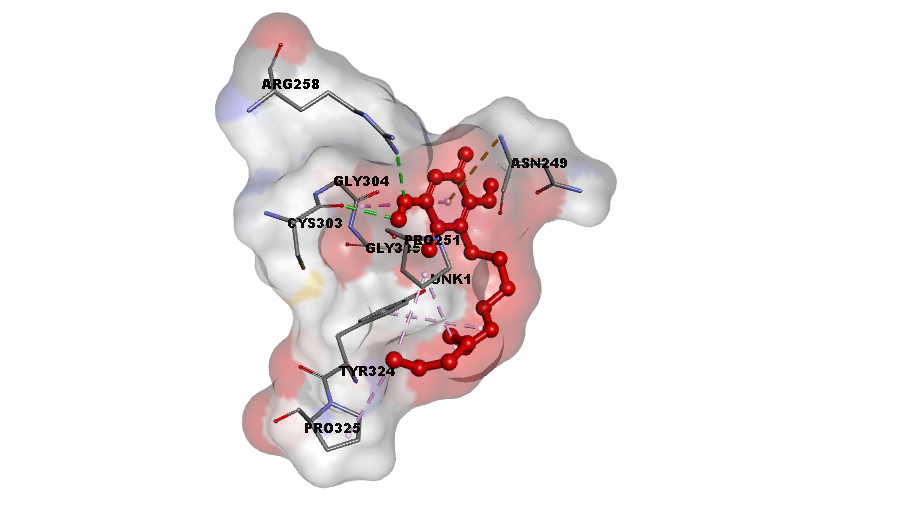** |
| **10281** | **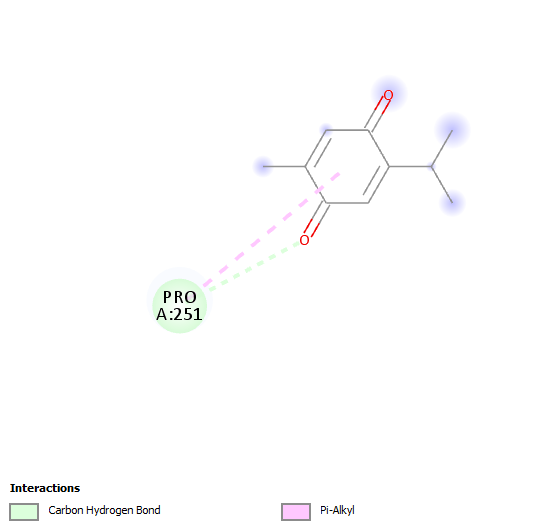** | **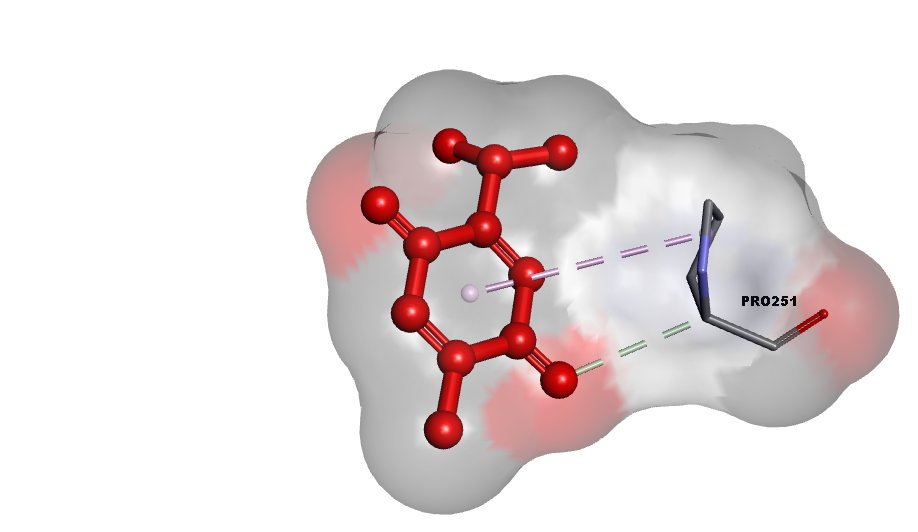** |
| **219100** | **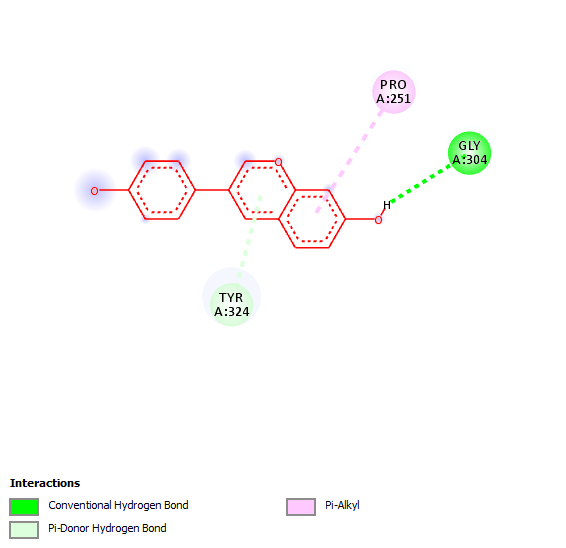** | **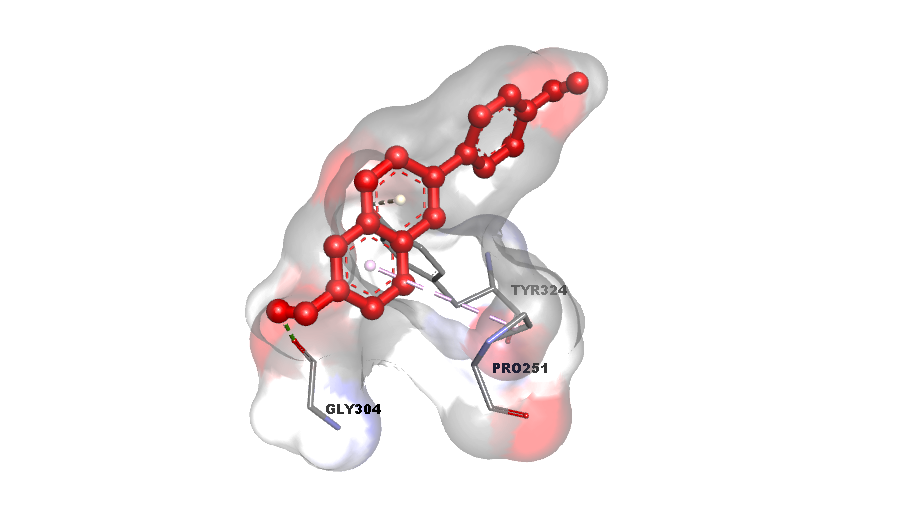** |
| **24737642** | **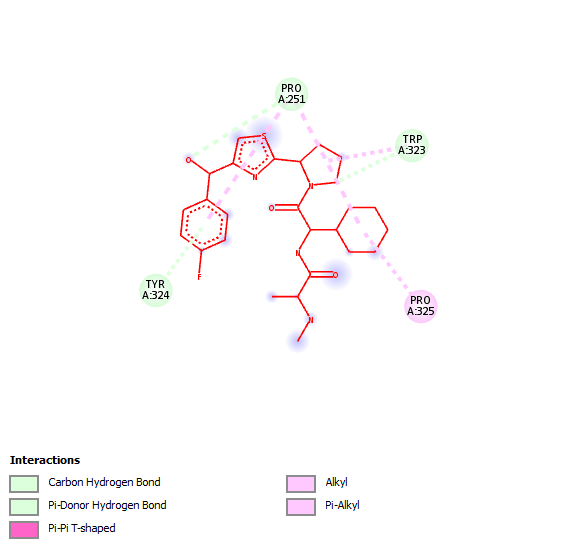** | **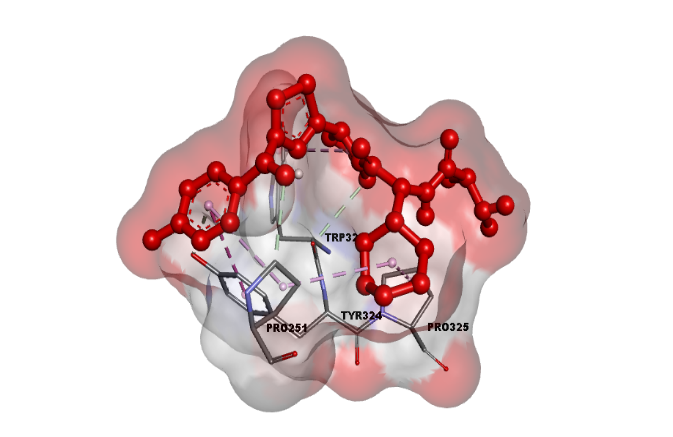** |
| **25022340** | **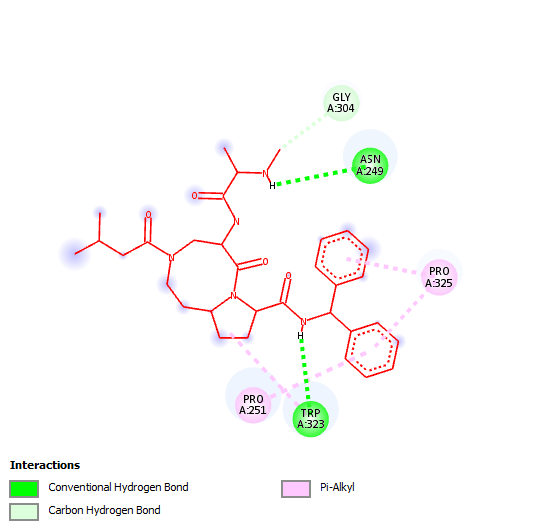** | **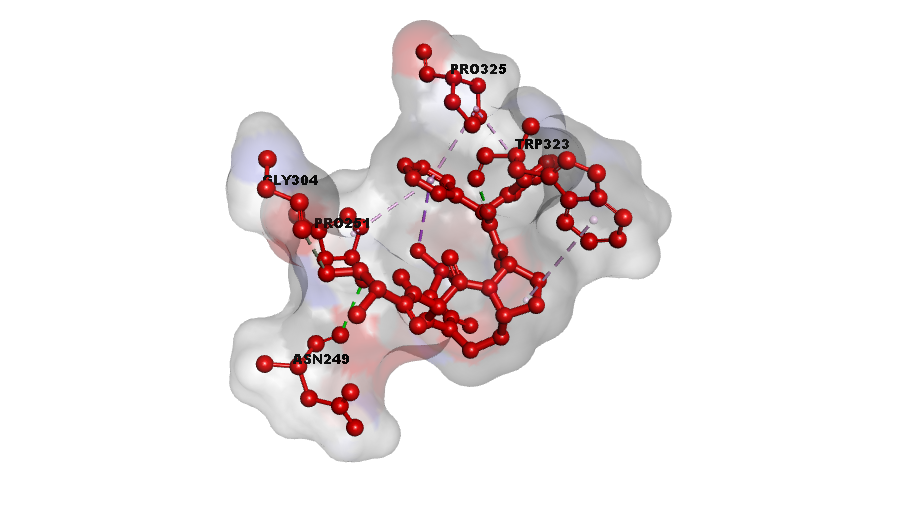** |
| **44182275** | **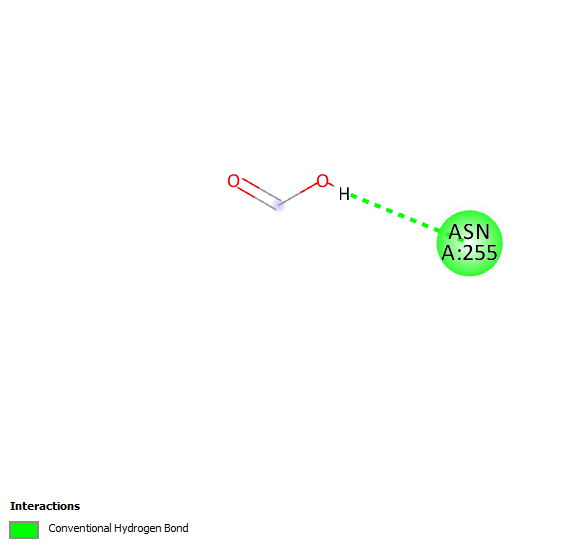** | **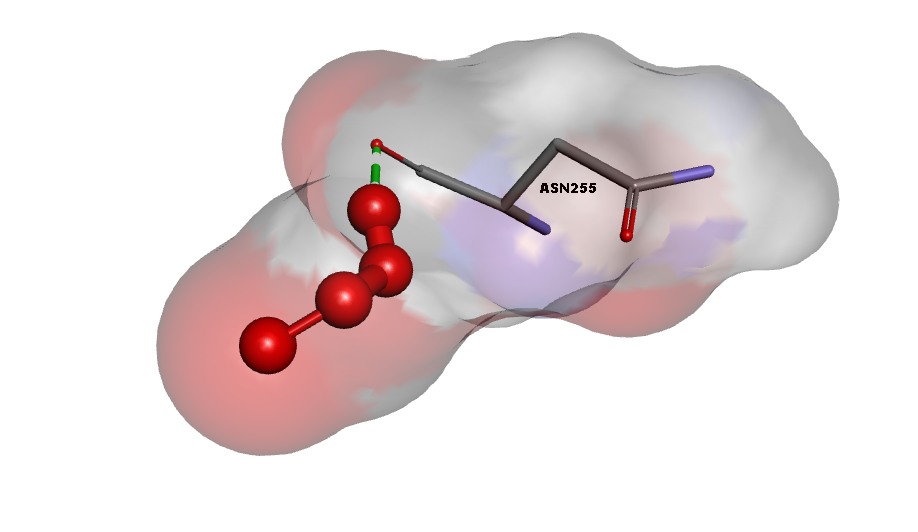** |
| **46781908** | **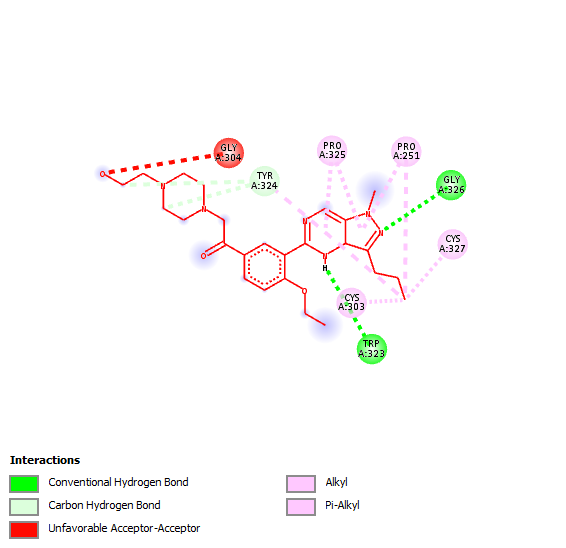** | **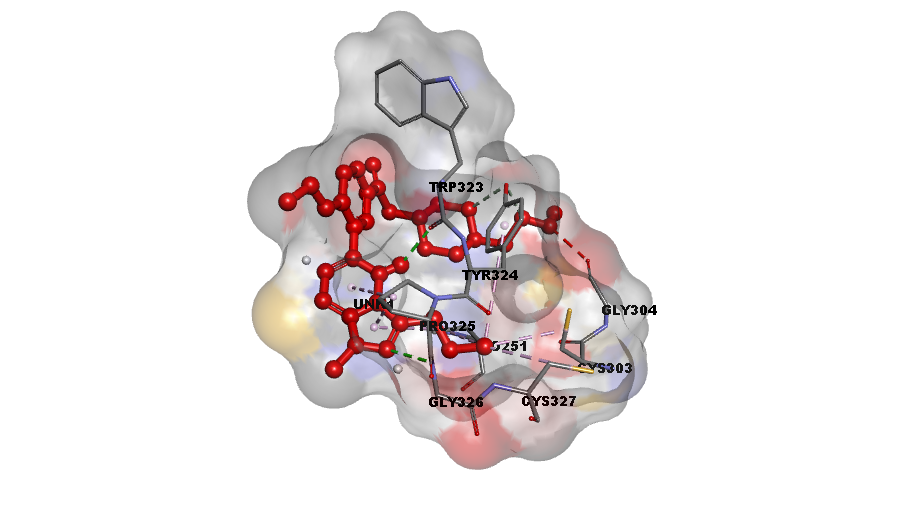** |
| **49836020** | **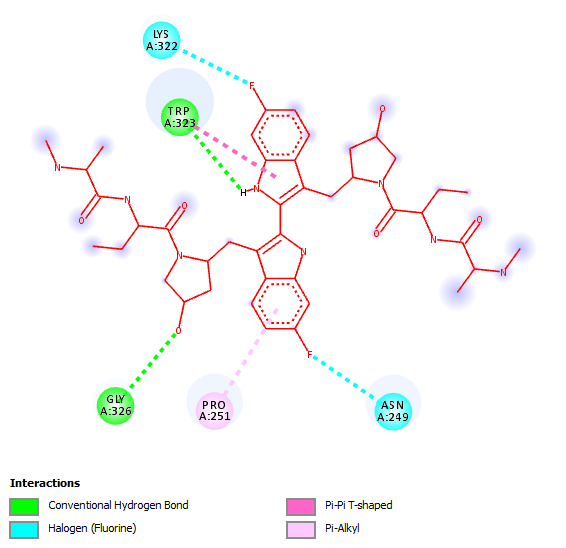** | **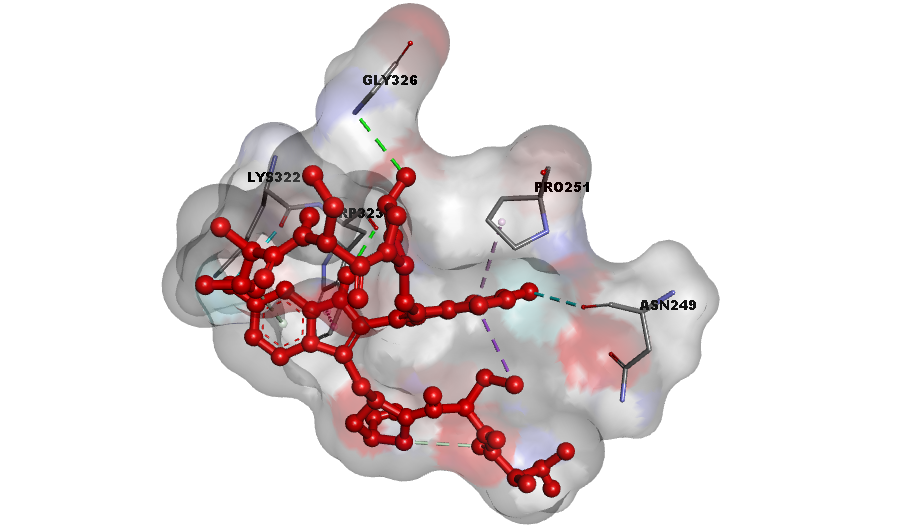** |
| **118169620** | **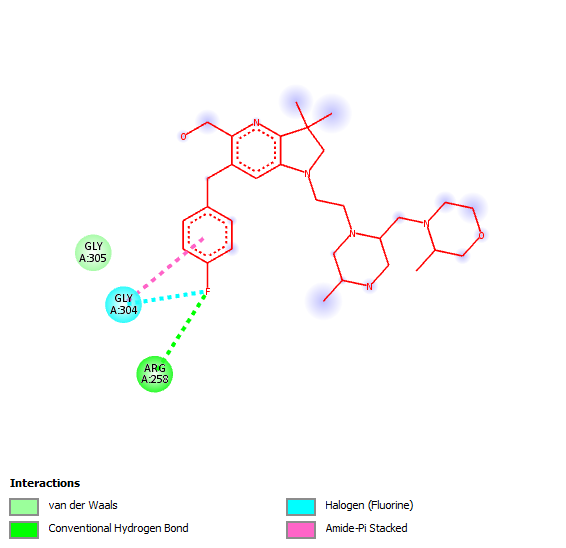** | **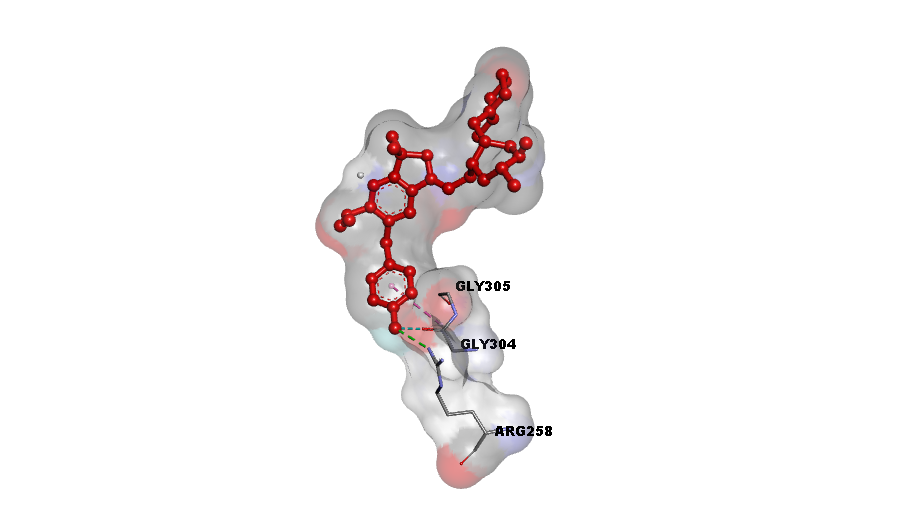** |

Table S2: Showing the docking score of selected 7 hit compounds with XIAP and Pharm-Fit Score found during pharmacophore-based screening process.

| **ZINC ID** | **Docking Score (kcal/mol)** | **Pharm-Fit Score** |
| --- | --- | --- |
| ZINC77257307 | -8.0 | 95.53 |
| ZINC1070004335 | -7.8 | 94.75 |
| ZINC247950187 | -7.6 | 95.70 |
| ZINC107434573 | -6.9 | 95.72 |
| ZINC14612168 | -6.4 | 94.91 |
| ZINC253388756 | -5.5 | 94.89 |
| ZINC253388755 | -5.2 | 94.62 |
